# Lymphangioleiomyomatosis: A Case Report and Review of Clinical Features and Management

**DOI:** 10.7759/cureus.8386

**Published:** 2020-06-01

**Authors:** Brooke E Kania, Swapna Jain, Brittanie West, Suzanne W Courtney

**Affiliations:** 1 Internal Medicine, United Hospital Center, Bridgeport, USA; 2 Family Medicine, United Hospital Center, Bridgeport, USA

**Keywords:** sporadic lymphangioleiomyomatosis, pneumothorax, cystic lung disease

## Abstract

Pulmonary sporadic lymphangioleiomyomatosis (LAM) is a female-dominant disease associated with smooth muscle cell proliferation, which results in cystic lung disease presenting commonly with dyspnea and pneumothorax. This article aims to present a patient with the common clinical features and complications of LAM so as to aid in the efficient diagnosis and treatment of future patients. Limited options in the management of LAM make early diagnosis key, as management focuses on supportive care to slow the progressive decline of pulmonary function. Workup includes a diagnosis of exclusion with specific antibodies or titers such as anti-Sjögren's syndrome type A (anti-SSA) antibodies, anti-Sjögren's syndrome type B (anti-SSB) antibodies, angiotensin-converting enzyme (ACE) levels, alpha-1-antitrypsin levels, and vascular endothelial growth factor (VEGF) antibodies with definitive diagnosis limited to tissue confirmation. Here, we discuss a 39-year-old female with dyspnea and spontaneous pneumothorax, who was subsequently diagnosed with LAM during her hospitalization and managed outpatient with sirolimus therapy.

## Introduction

Lymphangioleiomyomatosis (LAM) is a disorder affecting multiple systems, such as the kidney and lymphatics, with primary pathology involving the lung [1-2]. Patients who have LAM without tuberous sclerosis are considered to have sporadic LAM, which is caused by abnormal proliferation of smooth muscle cells [2]. Patients affected by LAM are primarily women of reproductive age who present with dyspnea, chest pain, coughing, or hemoptysis [2-3]. Management consists of supportive care, such as smoking cessation, providing influenza and pneumococcal vaccines, exercise, pulmonary rehabilitation, avoiding airplane travel, maintaining healthy weight and diet, as well as good psychosocial support [4-5]. Patients may receive sirolimus therapy to suppress respiratory decline and/or undergo pleurodesis to prevent complications such as pneumothorax [3-4]. Traditionally, LAM was managed via lung transplantation exclusively; however, with genetic testing and an increase in the patient study population, alternative management techniques are being researched [2].

This case report was earlier presented as a poster at the 2020 American College of Osteopathic Family Physicians Faculty Development/Program Director’s Workshop.

## Case presentation

A 39-year-old female presented to the emergency department with a chief complaint of two days of sharp, left-sided chest pain radiating to her left neck and left upper back associated with shortness of breath and an episode of presyncope. Past medical history was significant for chronic sinusitis and one episode of bronchitis, which was treated two months prior to her presentation. Family history was insignificant. The patient was never a smoker and lived a very healthy, active lifestyle. She worked as a sales representative for a technology company, requiring frequent travel across the country.

**Figure 1:**
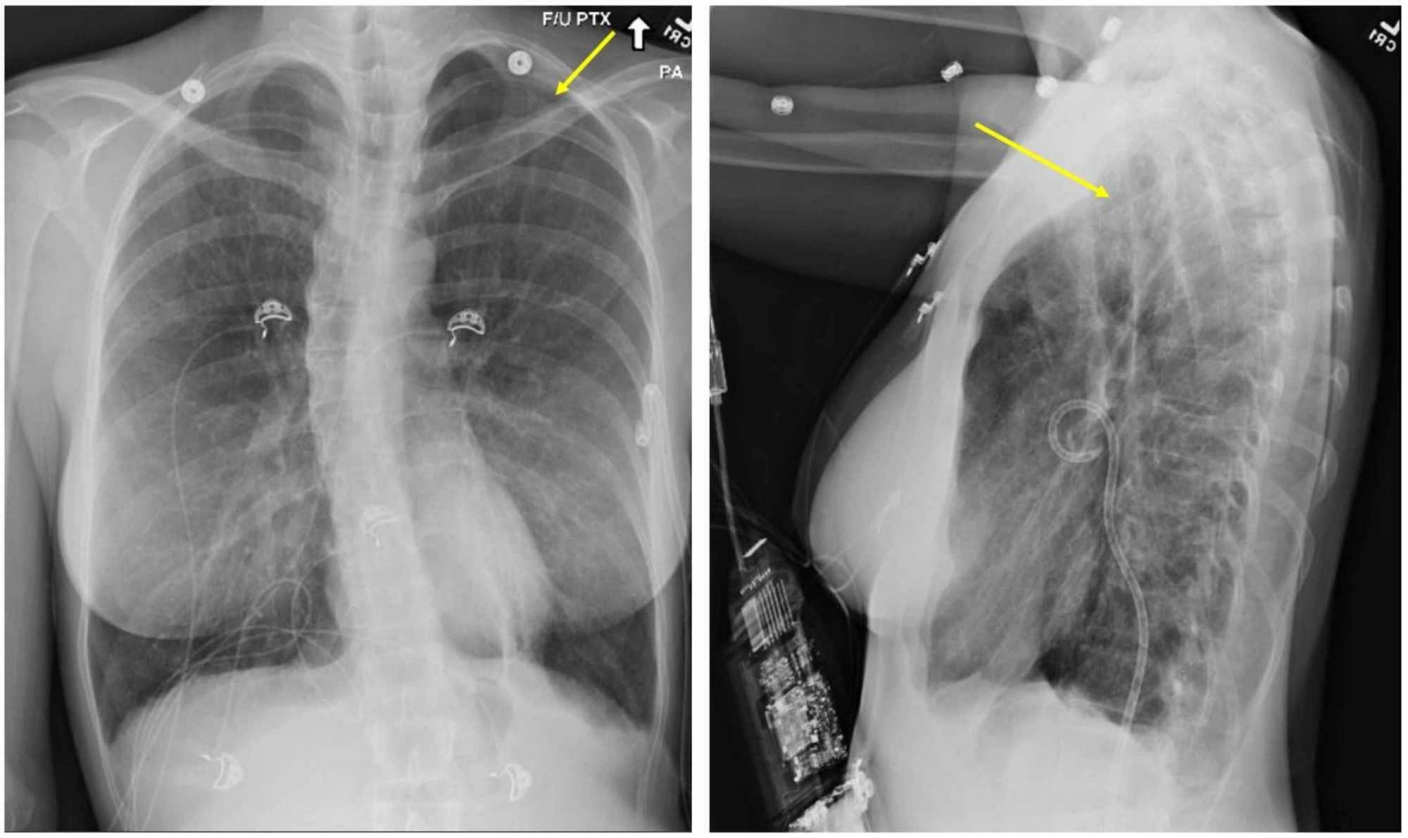
X-ray chest PA and lateral Left-sided pneumothorax PA: posteroanterior

**Figure 2:**
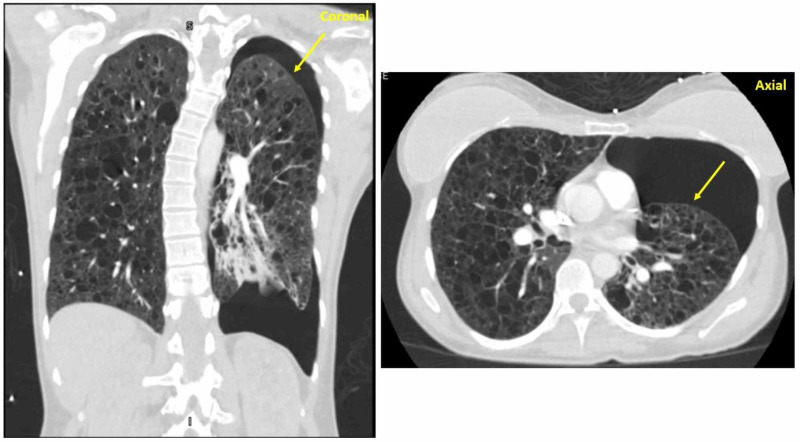
CT chest with IV contrast Coronal and axial views of left-sided pneumothorax shown with severe emphysema CT: computed tomography; IV: intravenous

Upon presentation to the emergency department, the patient appeared pale, diaphoretic, in respiratory distress, with an oxygen saturation of 91% on room air. The physical exam was significant for diffusely diminished lung sounds, especially in her left lung fields. Chest X-ray (Figure 1) and computed tomography (CT) chest (Figure 2) were significant for severe emphysema and large left pneumothorax requiring urgent pigtail thoracostomy. She was admitted with a left-sided chest tube and on nasal cannula oxygen for further management and workup of her spontaneous pneumothorax and severe emphysema. Pulmonology and cardiothoracic surgery were consulted for assistance in this case. Workup included a renal ultrasound negative for renal angiomyolipoma, negative human immunodeficiency virus (HIV) screen, no alpha-1-antitrypsin deficiency, normal angiotensin-converting enzyme (ACE) levels, and absent as anti-Sjögren's syndrome type A (anti-SSA) or as anti-Sjögren's syndrome type B (anti-SSB antibodies) but a mildly positive antinuclear antibody (ANA) titer of 1:40.

Due to the complexity of the patient’s case and suspected lymphangioleiomyomatosis (LAM), the patient was considered for transfer to a tertiary care center. However, insurance limited her transfer options, and in order to prevent a delay in care, the patient opted to proceed with surgical management during the current hospitalization. She underwent chemical pleurodesis to prevent further pneumothoraces with video-assisted thoracoscopic surgery (VATS) of the left lower lobe with wedge excision, which was sent to pathology, and pleural tent procedure to prevent air leaks postoperatively. She was extubated successfully onto nasal cannula oxygen and her postoperative course was uncomplicated.

**Figure 3:**
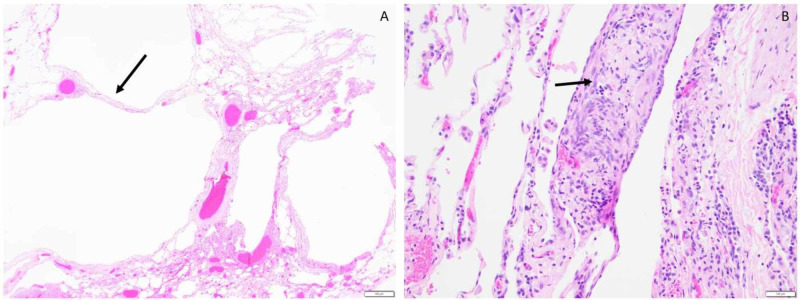
Left lower lobe lung with wedge excision A) Lung tissue with cystic change lined by thickened interstitium; B) Nodules of interstitial spindle cells, suggestive of lymphangioleiomyomatosis

**Figure 4:**
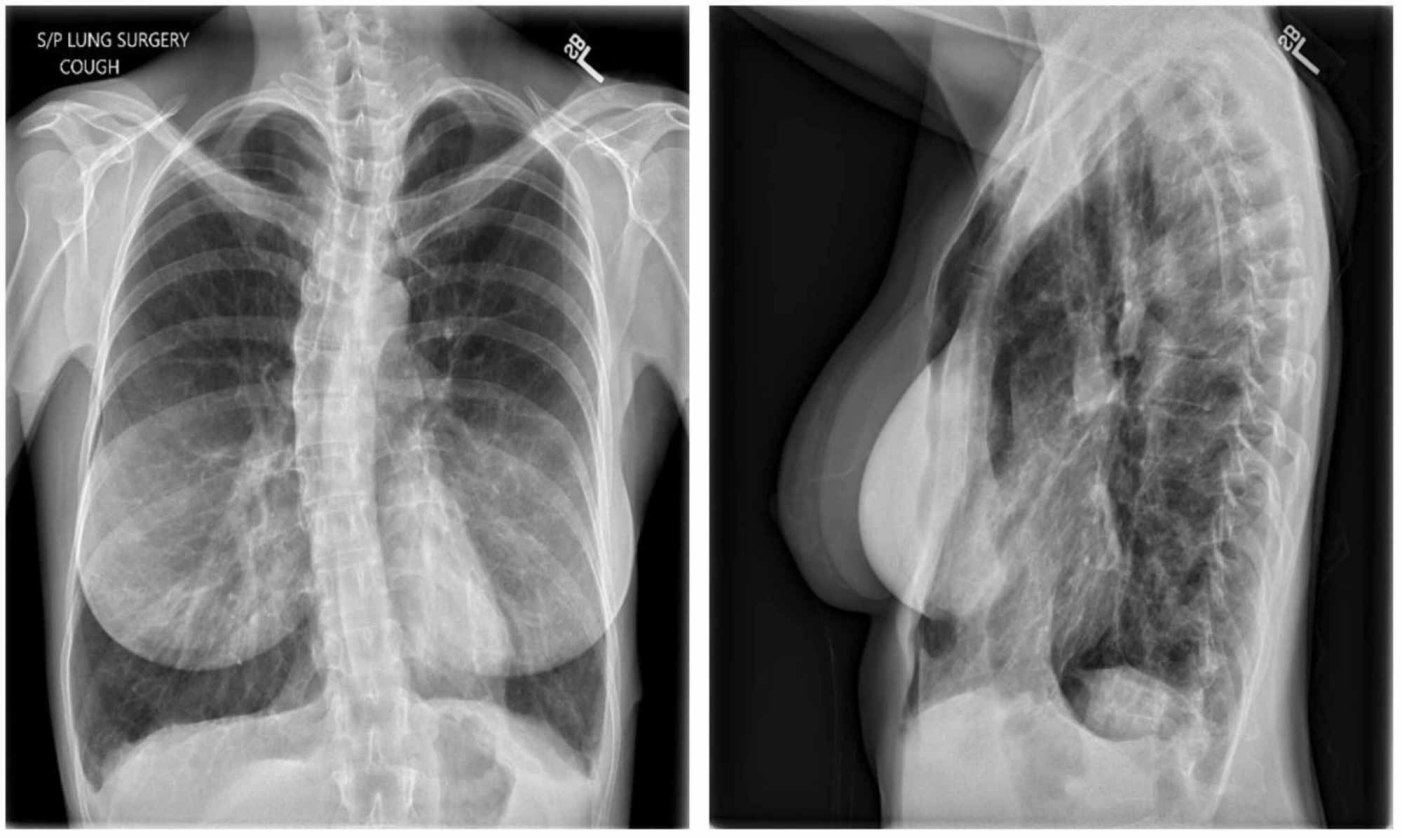
X-ray chest PA and lateral post-surgery No acute cardiopulmonary abnormality identified; chronic lung changes that are consistent with lymphangioleiomyomatosis PA: posteroanterior

Pathologic gross examination of the specimen was significant for lung parenchyma with prominent cystic changes and cystic structures coated by groups on bland epithelioid to spindle eosinophilic cells (Figure 3). Additional immunostaining revealed these cells were strongly positive for actin and progesterone receptors with significant human melanoma black (HMB45) and microphthalmia transcription factor (MiTF) staining. All of these features were pathologically consistent with the diagnosis of lymphangioleiomyomatosis. The patient was successfully discharged home on nasal cannula oxygen with follow-up appointments scheduled with pulmonology, cardiothoracic surgery, and the University of Pennsylvania's LAM specialty clinic. Postoperative instructions included limiting air travel, which was frequent as part of her career, to prevent future pneumothorax complications. As an outpatient, sirolimus therapy was initiated at the University of Pennsylvania, along with evaluation for bilateral lung transplantation in the future. Follow-up chest X-rays two months following hospital discharge showed chronic lung changes consistent with severe emphysematous disease and hyperinflation but were negative for pneumothorax (Figure 4).

## Discussion

Our patient presented with the classic demographic features of LAM, as she was a female in her childbearing years with pulmonary manifestations and a history of frequent flights. LAM is estimated to have an incidence of one case per 1,000,000 patients in the US and Europe; however, this disease is potentially underdiagnosed. LAM may be mistaken for other pulmonary disorders due to the similarities in the initial presenting symptoms, such as restrictive or obstructive lung diseases, and it may be inadequately treated with bronchodilators [1-2]. LAM patients who undergo exercise testing may exhibit hypoxemia with poor ventilation and gas exchange [3]. Pulmonary function testing may be significant for a decrease in forced expiratory volume in 1 second (FEV1) and diffusion capacity for carbon monoxide (DLCO) [4]. Radiographic findings can be notable for hyperinflated lungs and diffuse thin-walled cysts evident in lung parenchyma [4].

The pathophysiology of LAM includes the rapid expansion of smooth muscle cells in the lung parenchyma and airway walls, as well as the lymphatic system [3]. LAM has two specific types, sporadic and congenital. Sporadic cases comprise 60% of LAM patients, occurring later in life with fewer complications [6]. Congenital cases occur at younger ages in which patients have alterations in their tuberous sclerosis complex genes 1 and 2 (TSC1 and TSC2) [3]. TSC1 and TSC2 help regulate the mammalian target of rapamycin (mTOR) signaling pathways [7]. Congenital LAM presents as a more aggressive disease commonly affecting the brain, kidney, and integumentary systems, with less common pulmonary manifestations [8]. LAM cells morphologically consist of either myofibroblast spindle cells or epithelioid polygonal cells, which can proliferate and lead to alveolar air restriction [7]. Our patient presented as a sporadic LAM patient, as she was diagnosed as an adult and did not have TSC1 or TSC2 alterations present in her pathology. Patients may exhibit melanocytic mutation markers on biopsy, which can be relatively specific to pulmonary LAM. These markers include the HMB45 monoclonal antibody and MiTF, both of which stained positively in our patient’s biopsy [9-10].

Patients who present with LAM at a greater age and have a presence of angiomyolipoma associated with their disorders have been shown to have a decreased mortality risk [11]. LAM has become more manageable medically over the years with a median transplant-free survival rate of 29 years from the onset of symptoms [11]. Patients with this disease are at a greater risk of developing recurrent spontaneous pneumothoraces due to smooth muscle proliferation, airway narrowing, and alveolar damage due to cyst progression. Spontaneous pneumothoraces are estimated to occur in 10% of patients who have cystic lung diseases that are diffuse in nature [12]. Similar to our patient with LAM, patients who travel via airplane have a 3x increased risk of pneumothorax due to the possibility of subpleural cyst ruptures from changes in atmospheric pressure [13]. Patients with LAM who experience spontaneous pneumothorax have a higher risk of recurrence and, therefore, pleurodesis is a viable option to prevent further pneumothorax complications through lung re-expansion [12,14]. If in the future, patients require a lung transplant, prior pleurodesis management does not exclude them from eligibility [12]. If the patient has severe alveolar degeneration, the transplant may be viable when the DLCO is <40% of predicted and VO2max is <50% of predicted [15]. Pharmacologically, LAM patients can be treated with sirolimus, an immunosuppressant that targets mTOR and interrupts T-cell activation downstream of the interleukin-2 (IL-2) receptor. This treatment modality has had success in stabilizing pulmonary function in patients with LAM, as well as improving oxygenation to the lungs, exercise capability, and quality of life [14,16]. With therapeutic serum levels of sirolimus and medication compliance, this immune modulator can prevent further pneumothorax incidents [14].

## Conclusions

Lymphangiomyomatosis is a rare disease in young women that can present with spontaneous pneumothorax and dyspnea on exertion. The definitive treatment for this disease requires a lung transplant. If patients forgo transplant or are not transplant candidates, they can be managed pharmacologically with sirolimus. Recurrent pneumothoraces can be prevented with pleurodesis and avoidance of air travel. The goal of this case report is to improve the diagnosis of this condition by considering LAM as a differential diagnosis for reproductive-age female patients that present similarly to our patient presented in this case report. Subsequently, this should lead to further research and increased treatment options for LAM patients.

## References

[1] Yamazaki A, Miyamoto H, Futagawa T (2005). An early case of pulmonary lymphangioleiomyomatosis diagnosed by video-assisted thoracoscopic surgery. Ann Thorac Cardiovasc Surg.

[2] Hohman DW, Noghrehkar D, Ratnayake S (2008). Lymphangioleiomyomatosis: a review. Eur J Intern Med.

[3] Taveira-DaSilva AM, Moss J (2016). Epidemiology, pathogenesis and diagnosis of lymphangioleiomyomatosis. Expert Opin Orphan Drugs.

[4] Taveira-DaSilva AM, Steagall WK, Moss J (2006). Lymphangioleiomyomatosis. Cancer Control.

[5] McCormack FX, Gupta N (2020). Sporadic lymphangioleiomyomatosis: treatment and prognosis. UpToDate.

[6] Moss J, Avila NA, Barnes PM (2001). Prevalence and clinical characteristics of lymphangioleiomyomatosis (LAM) in patients with tuberous sclerosis complex. Am J Respir Crit Care Med.

[7] Krymskaya VP (2008). Smooth muscle-like cells in pulmonary lymphangioleiomyomatosis. Proc Am Thorac Soc.

[8] Riojas RA, Bahr BA, Thomas DB, Perciballi J, Noyes L (2012). A case report of lymphangioleiomyomatosis presenting as spontaneous pneumothorax. Mil Med.

[9] Martignoni G, Pea M, Reghellin D, Gobbo S, Zamboni G, Chilosi M, Bonetti F (2010). Molecular pathology of lymphangioleiomyomatosis and other perivascular epithelioid cell tumors. Arch Pathol Lab Med.

[10] Tanaka H, Imada A, Morikawa T, Shibusa T, Satoh M, Sekine K, Abe S (1995). Diagnosis of pulmonary lymphangioleiomyomatosis by HMB45 in surgically treated spontaneous pneumothorax. Eur Respir J.

[11] Oprescu N, McCormack FX, Byrnes S, Kinder BW (2013). Clinical predictors of mortality and cause of death in lymphangioleiomyomatosis: a population-based registry. Lung.

[12] Cooley J, Gary Lee YC, Gupta N (2017). Spontaneous pneumothorax in diffuse cystic lung diseases. Curr Opin Pulm Med.

[13] Gonano C, Pasquier J, Cécile Daccord (2018). Air travel and incidence of pneumothorax in lymphangioleiomyomatosis. Orphanet J Rare Dis.

[14] Zhou L, Ouyang R, Luo H (2018). Efficacy of sirolimus for the prevention of recurrent pneumothorax in patients with lymphangioleiomyomatosis: a case series. Orphanet J Rare Dis.

[15] Taveira-DaSilva AM, Stylianou MP, Hedin CJ (2003). Maximal oxygen uptake and severity of disease in lymphangioleiomyomatosis. Am J Respir Crit Care Med.

[16] Hu S, Wu X, Xu W (2019). Long-term efficacy and safety of sirolimus therapy in patients with lymphangioleiomyomatosis. Orphanet J Rare Dis.

